# Improved surgical accuracy in total knee arthroplasty using the ROSA® knee system: A randomised‐controlled unblinded trial

**DOI:** 10.1002/ksa.70197

**Published:** 2025-12-01

**Authors:** Alexander Möller, Feras Kasabji, Maximilian Fischer, Janosch Schoon, André Hofer, Georgi Iwan Wassilew, Johannes Christian Reichert

**Affiliations:** ^1^ Center for Orthopedics, Trauma Surgery and Rehabilitation Medicine University Medicine Greifswald Greifswald Germany

**Keywords:** clinical outcome, limb alignment, PROM, robotic‐assisted total knee arthroplasty, total knee arthroplasty

## Abstract

**Purpose:**

Robotic‐assisted total knee arthroplasty (RATKA) has been connected to improved surgical precision and implant alignment compared to conventional TKA (CTKA), with limited evidence from randomised‐controlled trials (RCT) and a large range of different systems on the market. Consequently, this study aimed to compare RATKA using the ROSA® knee system and CTKA in a prospective RCT for surgical as well as patient‐reported outcomes.

**Methods:**

Seventy‐seven patients undergoing TKA were prospectively included in this monocentric, unblinded RCT. All patients had a minimum follow‐up of 1 year. Primary endpoints were postoperative implant alignment accuracy and patient‐reported outcomes using the Oxford knee score (OKS) and quality of life scoring (EQ‐5D‐5L). Secondary outcomes included range of motion, blood loss, length of stay, return to work and physiotherapy needs. Statistical analysis included multivariable regression with significance set at *p* < 0.05.

**Results:**

RATKA lead to improved limb alignment with significant lower frequency of extreme malalignment (>3°; <−3° from the mechanical axis) compared to CTKA (38% vs. 18%, *p* = 0.047). Both groups had comparable postoperative improvement in patient‐reported outcomes (OKS Δ prepost 18.0 vs. 17.7, *p* = 0.902; EQ‐5D‐5L Δ prepost 0.21 vs. 0.25, *p* = 0.089). Regression analysis identified preoperative OKS (*b* = 0.57, *p* < 0.001) and cruciate‐retaining implant bearing (*b* = 5.42, *p* = 0.043) as significant predictors of improved postoperative outcome.

**Conclusions:**

RATKA using the ROSA® knee system improves implant positioning and radiological alignment compared to CTKA with similar short‐term clinical outcomes. Future research should focus on long‐term RCT data to determine whether the improved accuracy of RATKA results in superior implant survival and reduced revision rates.

**Level of Evidence:**

Level II, prospective comparative study.

**Abbreviations**
ANOVA: analysis of Variance
BMI: body mass index
CI: confidence interval
CR: cruciate‐retaining
CTKA: conventional total knee arthroplasty
Hb: haemoglobin
HKAA: hip knee ankle angle
IEC: independent ethics committee
IQR: interquartile range
OKS: Oxford knee score
PROMs: patient‐reported outcome measures
PS: posterior‐stabilised
RATKA: robotic‐assisted total knee arthroplasty
RCT: randomised controlled trial
SD: standard deviation
TKA: total knee arthroplasty
VAS: visual analogue scale score

## INTRODUCTION

Total knee arthroplasty (TKA) is a successful and safe surgical procedure, with patient satisfaction rates ranging from 70% to 85% of cases [3, 5]. To further improve therapeutic outcomes in conventional total knee arthroplasty (CTKA), substantial efforts have been made to develop various assistive technologies including navigated TKA and robotic‐arm assisted (RATKA).

Several case series have already associated RATKA with improved implant positioning and more accurate translation of the planned alignment into the final surgical outcome [10, 19]. Additionally, robotic assistance may reduce soft‐tissue damage during surgery [11]. Furthermore, there are conflicting results regarding patient‐reported outcomes comparing RATKA and CTKA. While some studies demonstrate superior clinical outcomes for RATKA [26], others report no difference [21].

These conflicting results regarding postoperative outcomes could potentially be driven by the wide range of robotic systems available on the market. Furthermore, there is paucity in literature regarding radiographic and clinical outcomes from high‐quality randomised‐controlled trials (RCTs). In this respect, no all‐encompassing RCT with short‐term follow‐up has focused on the frequently used ROSA® knee system (ZimmerBiomet), a robotic platform that assists the surgeon through active guidance in jig positioning [4].

Consequently, the present study aimed to compare radiographic and clinical outcomes for RATKA using the ROSA® knee system (ZimmerBiomet) and CTKA in an RCT, further employed by multivariable linear regression to adjust for important covariates such as implant type, patient demographics, baseline function and surgical characteristics. It was hypothesised that RATKA using the ROSA® knee system led to improved accuracy in achieving a precise postoperative total limb alignment and superior patient‐reported functional outcomes compared to CTKA.

## METHODS

### Study design

Seventy‐seven patients (78 knees) undergoing TKA for primary knee osteoarthritis were prospectively enrolled in this monocentric, randomised controlled trial between July 2022 and November 2023. All patients had a minimum follow‐up of 1 year and gave written‐informed consent before study enrolment. Ethics approval was obtained from the local independent ethics committee (IEC) according to the World Medical Association Declaration of Helsinki.

**Figure 1:**
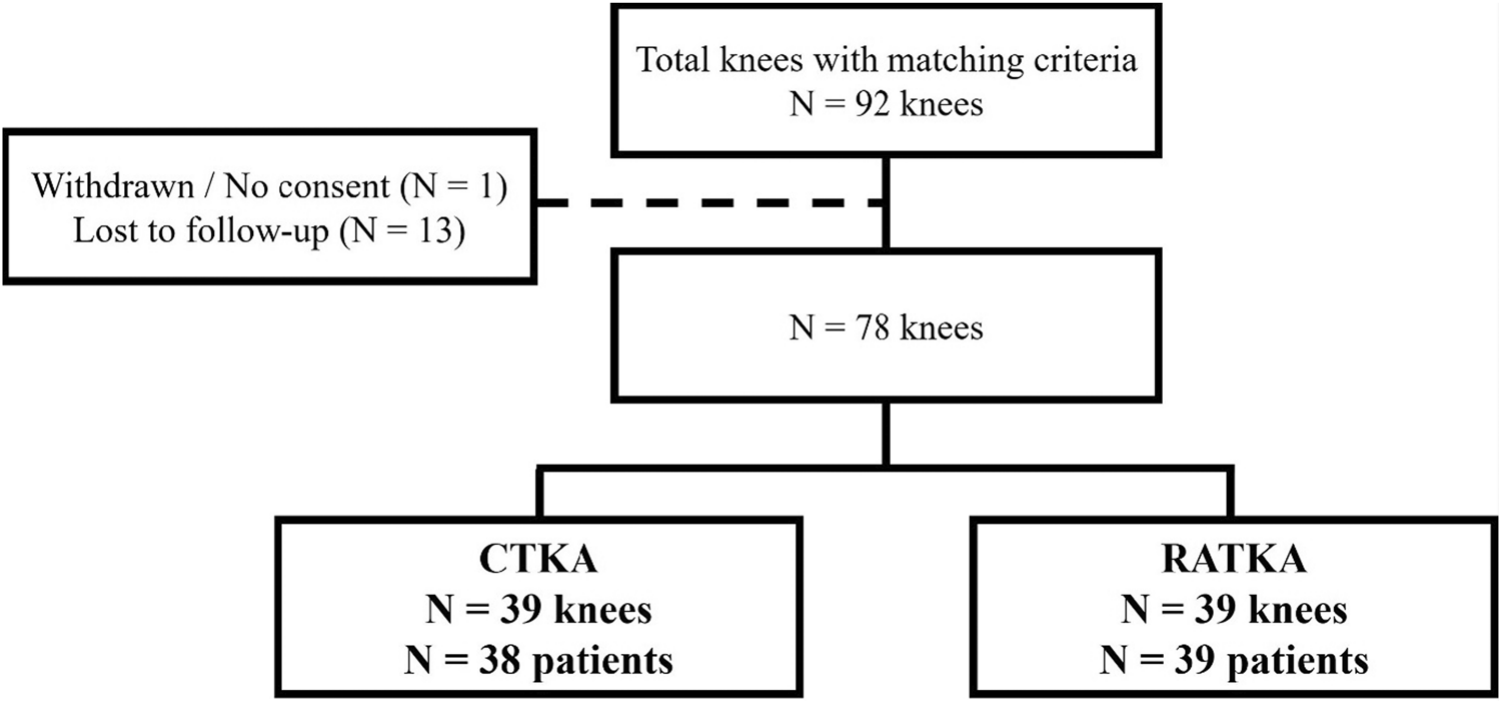
Study cohort.

Patients were randomly assigned to the equally contributed groups (*n* = 39) by a separate surgical coordinator, not involved in the current study and unaware of patient profiles or medical records. Both groups were compared for TKA outcomes: Group I—conventional jig‐based TKA (CTKA), Group II—RATKA with imageless intraoperative robotic planning and assistance of the ROSA® knee system (ZimmerBiomet). Exclusion criteria were severe systemic diseases (ASA ≥ 3), contralateral knee or hip osteoarthritis (Kellgren andLawrence grade >3), radicular back pain, osteoporosis and posttraumatic osteoarthritis. Patients could not choose their treatment allocation and were preoperatively counselled regarding both treatment options. This study was not blinded, both patients and surgeon were aware of the surgical group assignment. Both groups were examined preoperatively as well as at 12‐month follow‐up. A flowchart of the study cohorts including withdrawn consent and lost to follow‐up are shown in Figure 1.

### Surgical technique

All surgical procedures were performed by a single, fellowship‐trained high‐volume surgeon, using a medial parapatellar approach following a restricted kinematic alignment implantation strategy [15]. The implants used for this study were the Persona® TKA (ZimmerBiomet) with a cruciate‐retaining (CR) or a posterior‐stabilised (PS) bearing. Criteria for PS bearing were an insufficient posterior cruciate ligament, excessive posterior rollback, instability during knee flexion, contractures or severe valgus or varus deformity and limited room of motion (knee flexion and extension). Otherwise, a CR bearing was used. Patients' choices were not considered. Both the femoral and tibial implant components were cemented.

### Outcome parameters

All patients completed a standardised assessment preoperatively and at 12‐month follow‐up. The primary outcome was set on the technical accuracy achieving the native leg alignment postoperatively and functional outcome after TKA using the Oxford knee score (OKS) [7, 8] and health‐related quality of life scores (EQ‐5D‐5L) [14]. Secondary outcomes were the knee range of motion, surgical time, intraoperative blood loss as measured by the reduction in postoperative haemoglobin levels (1st postoperative day vs. preoperative), length of stay, timing of return to work and number of postoperative physiotherapy units after hospital discharge.

### Data analysis

Statistical analyses were performed using Python (version 3.13.3) and GraphPad Prism (version 10.2.3, GraphPad Software, Inc.). Data loading, cleaning and data manipulation were conducted using the ‘Pandas’ library (version 2.3.0). Numerical operations were done by NumPy (version 2.3.0) [12].

Descriptive statistics were computed for all relevant variables to summarise patient characteristics and short‐term outcome measures. Normality of continuous data distributions was assessed using the Shapiro–Wilk test from the scipy.stats module [28].

For comparisons between independent groups, parametric *T*‐tests were conducted for normally distributed continuous variables. When assumptions for these tests were violated, nonparametric Mann–Whitney *U* tests were used. Associations between categorical variables were examined using chi‐squared tests. These tests were all performed using the scipy.stats module [28].

Given the multifactorial nature of postoperative outcomes a multivariable linear regression model was used to examine the relationships of OKS differences between preoperative and postoperative measurements as an indicator of improvement and patient satisfaction, and whether the surgery was robotic‐assisted or not, while controlling for age, sex, body mass index (BMI), bearing type (PS vs. CR), surgical time, length of stay and number of postoperative physiotherapy units. The preoperative OKS served as a baseline measurement. To reduce the risk of overfitting given the limited sample size, a linear regression with a reduced set of covariates (robotic assistance, age, sex, baseline OKS, implant type) was performed. A full model including all variables was run as a sensitivity analysis.

These models were implemented using the statsmodels library (version 0.14.4) [24]. Model assumptions including linearity, independence of errors, homoscedasticity and normality of residuals were assessed using visual inspection of residual plots as well as VIF comparisons and the Shapiro–Wilk test. All statistical tests were two‐tailed, and a significance level of *α* = 0.05 was adopted.

Data visualisation was generated using Graphpad Prism as well as Python′s Matplotlib (version 3.10.3) [18] and seaborn (version 0.13.2) [29].

## RESULTS

A total of 78 knees from 77 patients undergoing TKA were included in the study. Preoperative patients' characteristics were comparable between both groups (Table 1). The median follow‐up was 12.74 months (CTKA 13.02 months, RATKA 12.36 months). Preoperative coronal alignments were included and grouped into normal, deviant and aberrant [16].

**Table 1:** Patient characteristics and preoperative joint morphology.

	CTKA	RATKA	*p*
Age (years) (mean with SD)	67.0 ± 8.8	68.7 ± 9.3	0.407
Sex (% female)	53.8	64.1	0.357
BMI (kg/m^2^) (mean with SD)	33.6 ± 7.9	32.2 ± 6.3	0.543
Preoperative hip knee ankle angle (degrees) (mean with SD)	7.5 ± 4.1	7.0 ± 4.4	0.673
Knee phenotype (%)	Normal: 85.7	Normal: 85.3	1.000
	Deviant: 5.7	Deviant: 5.9	1.000
	Aberrant: 8.6	Aberrant: 8.8	1.000

*Note*: Normally distributed data: unpaired *t*‐test, mean ± SD; nonnormally distributed data: Mann–Whitney test, median and IQR.

Abbreviations: BMI, body mass index; CKTA, conventional total knee arthroplasty; IQR, interquartile range; RATKA, robotic‐assisted total knee arthroplasty.

**Figure 2:**
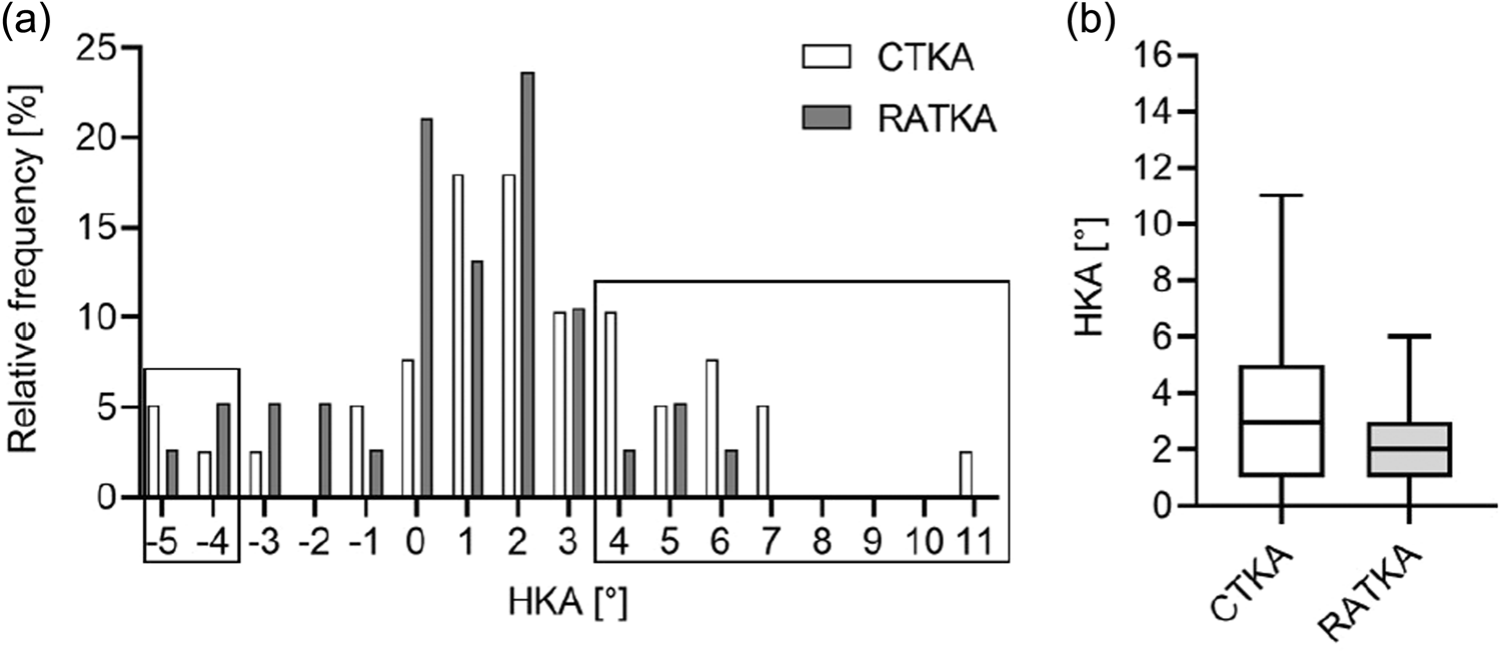
(a) Relative frequency of hip knee ankle angle (CTKA *n* = 39, RATKA *n* = 38), (b) hip knee ankle angle (CTKA *n* = 39, RATKA *n* = 38) (normally distributed data: unpaired *t*‐test, mean ± SD; nonnormally distributed data: Mann–Whitney test, Tukey boxplots). CKTA, conventional total knee arthroplasty; RATKA, robotic‐assisted total knee arthroplasty.

Postoperatively, radiographic analysis showed a significant lower frequency of extreme alignment deviation (>3°; <−3° from the mechanical axis) in the RATKA group (CTKA 38.5%, RATKA 17.9%, *p* = 0.047) (Figure 2a), with comparable postoperative HKAA in both groups (CTKA 3°, RATKA 2°, *p* = 0.067) (Figure 2b). All postoperative coronal alignments were classified as normal [16].

**Figure 3:**
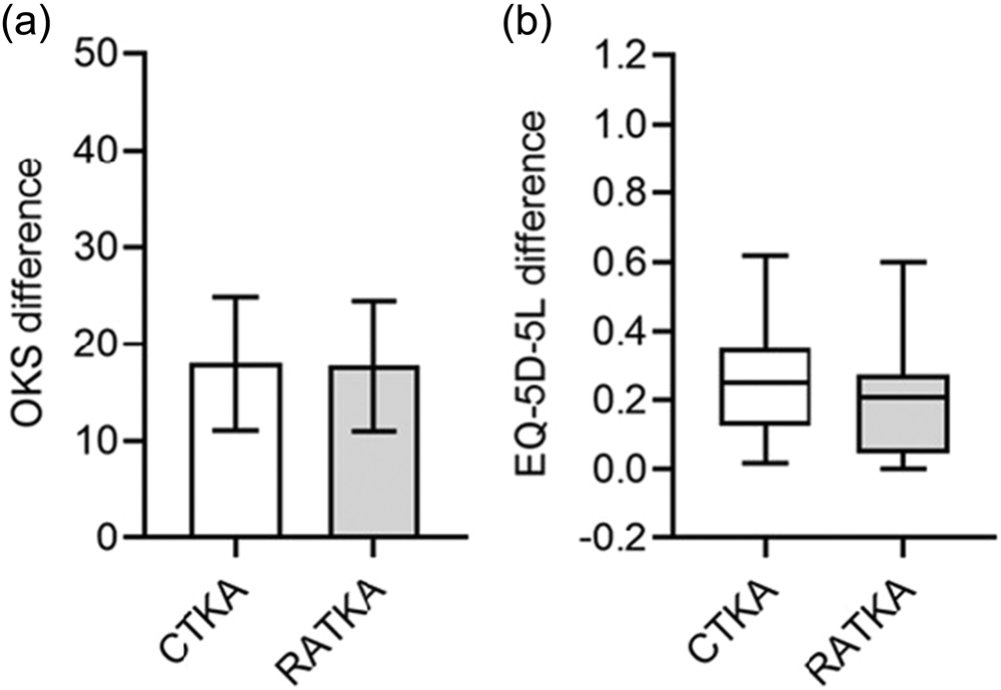
(a) Oxford knee score (OKS) difference (conventional total knee arthroplasty [CTKA] *n* = 27, robotic‐assisted total knee arthroplasty [RATKA] *n* = 26), (b) EQ‐5D‐5L difference (CTKA *n* = 25, RATKA *n* = 26) (normally distributed data: unpaired *t*‐test, mean ± SD; nonnormally distributed data: Mann–Whitney test, Tukey boxplots).

There was a significant increase in OKS at the latest follow‐up for both groups (Δ pre‐post CTKA 18.0, RATKA 17.7, *p* < 0.001), without significant intergroup difference (*p* = 0.902) (Figure 3a). In addition, there was no significant intergroup difference for postoperative improvement in EQ‐5D‐5L at the latest follow‐up (CTKA 0.25, RATKA 0.21, *p* = 0.089) (Figure 3b).

**Figure 4:**
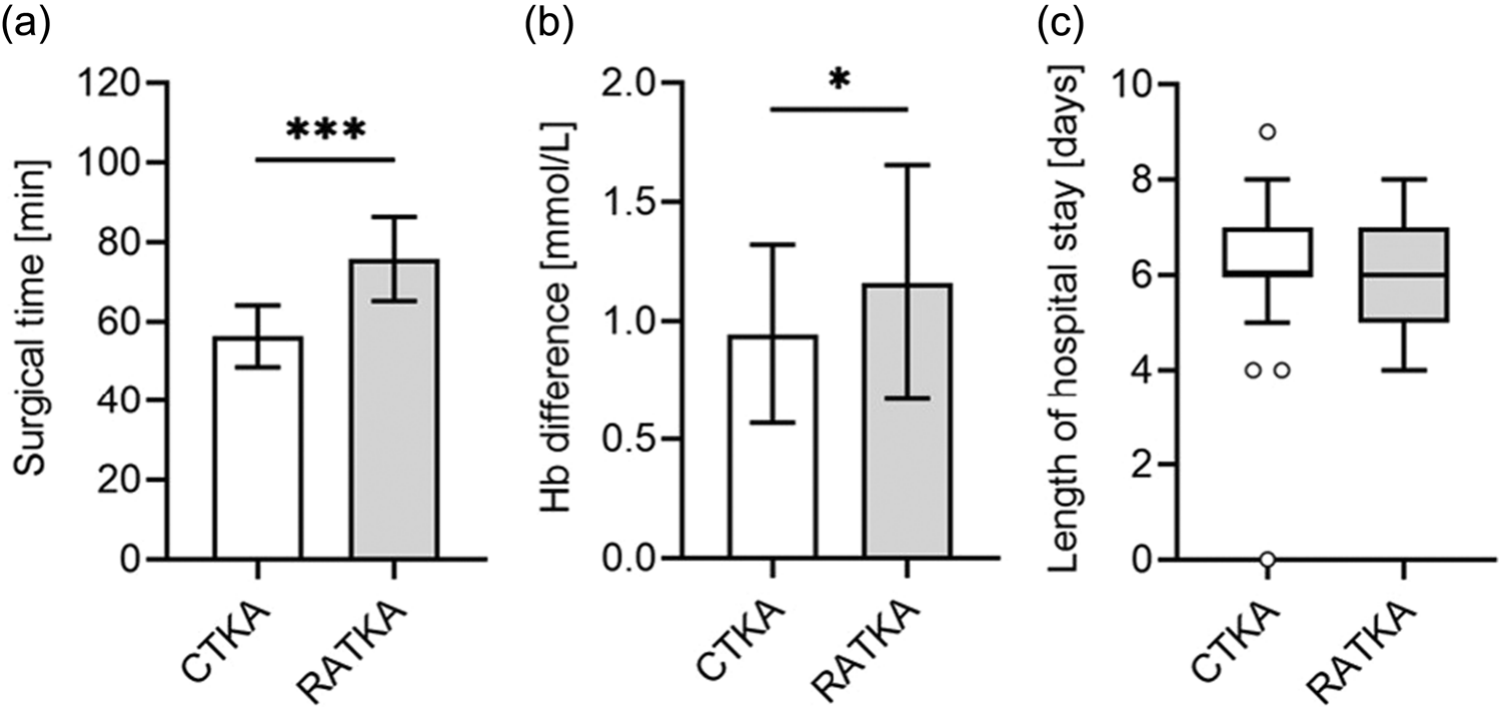
(a) Surgical time (each cohort *n* = 39), (b) reduction in postoperative haemoglobin levels (each cohort *n* = 39), (c) length of stay (each cohort *n* = 39) (normally distributed data: unpaired *t*‐test, mean ± SD; nonnormally distributed data: Mann–Whitney test, Tukey boxplots; *p < 0.05, ***p < 0.001).

Secondary outcomes showed no difference in time to return to work (CTKA 150 days, RATKA 157 days, *p* = 0.794) and both groups received a similar amount of physiotherapy postoperatively, with a median of 12 sessions each (Table 2). However, the surgical time was significantly longer in the RATKA cohort (CTKA 56.3 min, RATKA 75.8 min, *p* < 0.001) (Figure 4a), accompanied by a significant higher blood loss (Δ pre‐post CTKA −0.9 mmol/l, RAKTA −1.2 mmol/l, *p* = 0.029) (Figure 4b). Furthermore, no difference was found in length of hospital stay with a median time of 6 days in both cohorts (*p* = 0.143) (Figure 4c).

**Table 2:** postoperative results.

	CTKA	RATKA	*p*
Hip knee ankle angle (degrees) (median with SD)	3 (3.5)	2 (2)	0.067
Outliers of hip knee ankle angle (proportion) [%]	38	18*	0.047
Knee phenotype (%)	Normal: 100	Normal: 100	1.000
	Deviant: 0.0	Deviant: 0.0	1.000
	Aberrant: 0.0	Aberrant: 0.0	1.000
Improvement of OKS (mean with SD)	18.0 ± 7.0	17.7 ± 6.7	0.902
Improvement of EQ‐5D‐5L (median and IQR)	0.25 (0.21)	0.21 (0.20)	0.089
Time to return to work (days) (median and IQR)	150 (90.50)	157 (34.50)	0.794
Units of physiotherapy (median and IQR)	12 (18)	12 (18)	0.754
Surgical time (minutes) (mean with SD)	56.3 ± 7.8	75.8 ± 10.6***	<0.001
Reduction in haemoglobin levels (mmol/l) (mean with SD)	0.9 ± 0.4	1.2 ± 0.5*	0.029
Length of stay (days) (median and IQR)	6.00 (1.00)	6.00 (1.00)	0.143
Knee flexion (degrees) (median and IQR)	120 (20)	125 (30)	0.991
Knee extension deficit (degrees) (median and IQR)	0 (0)	0 (4)*	0.037

*Note*: Normally distributed data: unpaired *t*‐test, mean ± SD; nonnormally distributed data: Mann–Whitney test, median and IQR.

Abbreviations: CKTA, conventional total knee arthroplasty; IQR, interquartile range; OKS, Oxford knee score; RATKA, robotic‐assisted total knee arthroplasty.

* *p* < 0.05

*** *p* < 0.001.

**Figure 5:**
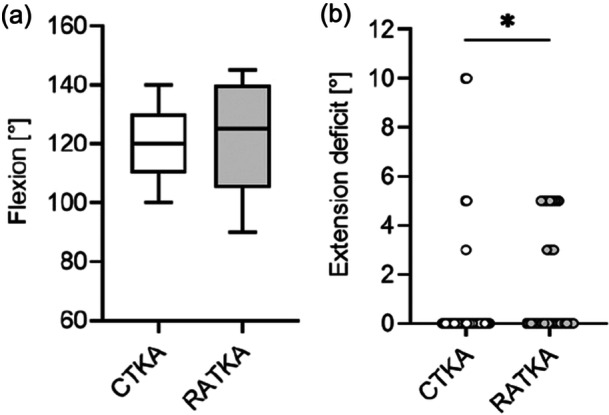
(a) Knee flexion (conventional total knee arthroplasty [CTKA] *n* = 35, robotic‐assisted total knee arthroplasty [RATKA], *n* = 37), (b) knee extension deficit (CTKA *n* = 35, RATKA *n* = 37) (normally distributed data: unpaired *t*‐test, mean ± SD; nonnormally distributed data: Mann–Whitney test, Tukey boxplots; *p < 0.05).

There was no significant intergroup difference in postoperative knee flexion for each cohort (CTKA 120°, RATKA 125°, *p* = 0.991) (Figure 5a). Knee extension deficit was also comparable, with a median of 0° in both cohorts (*p* = 0.037). However, there were fewer outliers among CTKA, so the difference was significant (Figure 5b).

### Linear regression analysis

The multiple linear regression model accounted for approximately 40% of the variance in OKS difference (adjusted *R*² = 0.36). Among the included covariates, preoperative OKS showed a significant positive association with postoperative OKS (*b* = 0.57, *p* < 0.001), indicating that better preoperative knee function was associated with higher postoperative OKS. Implant type (PS vs. CR) was also significantly associated with postoperative OKS (*b* = –5.42, *p* = 0.043), suggesting that the PS implant type was linked to a lower postoperative OKS compared to CR. Although not reaching statistical significance, RATKA showed a trend to a positive association with the postoperative OKS (*b* = 2.78, *p* = 0.13).

**Figure 6:**
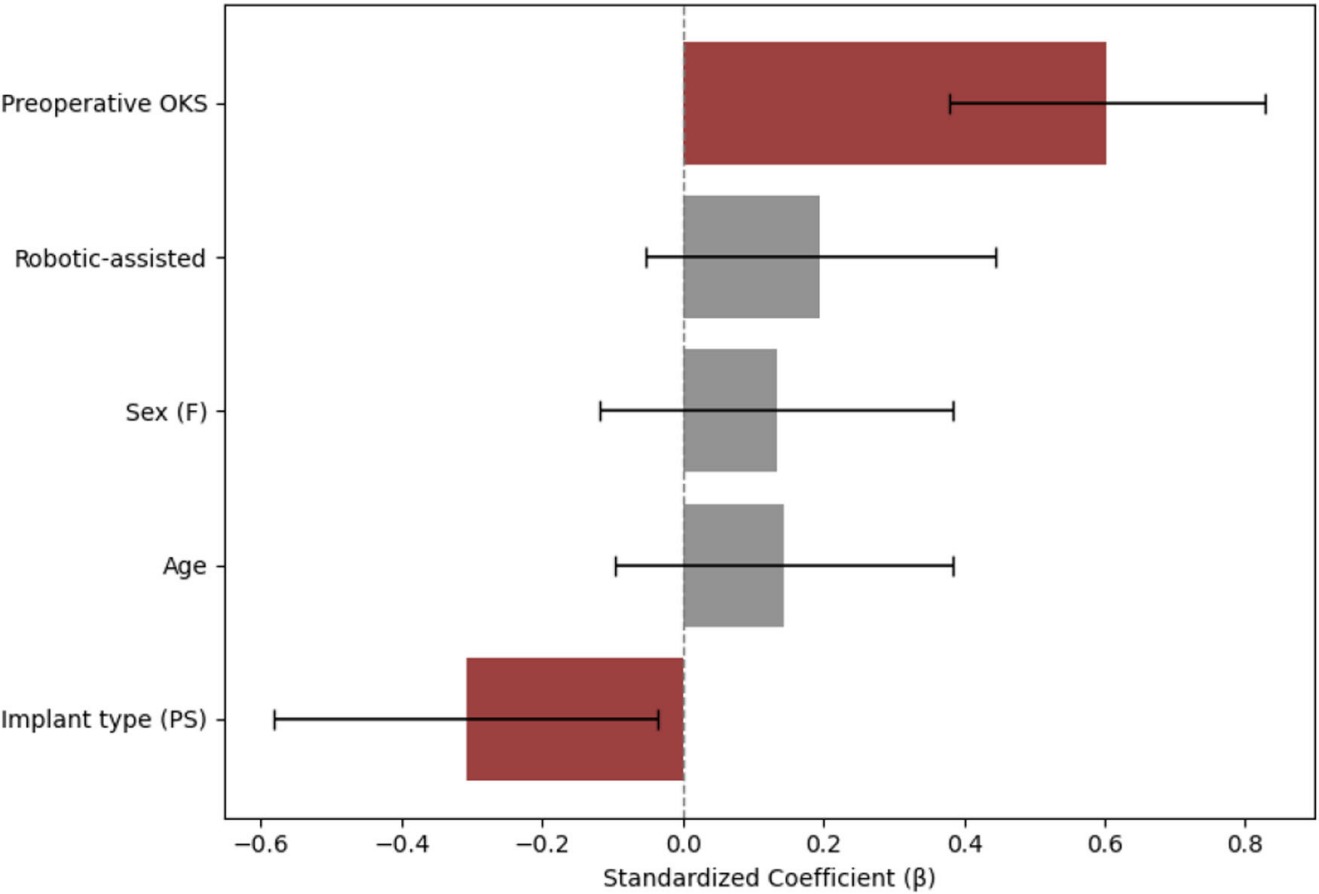
Factors associated with postoperative Oxford knee score (OKS) (standardised coefficients).

Other covariates, including sex and age did not demonstrate significant associations with postoperative OKS (all *p* > 0.05). Further covariates of the full model (BMI, surgical time, length of hospital stay and number of physiotherapy units) were tested and no significant associations were found (all *p* > 0.05). All results of the reduced model were consistent in the full model (adjusted *R*² = 0.39) (Figure 6).

## DISCUSSION

The main findings of this randomised‐controlled study were: (I) RATKA performed with the ROSA® knee system resulted in similar patient‐reported outcomes compared to CTKA; and (II) overall surgical accuracy is improved in RATKA leading to precise postoperative limb alignment with reduced frequency of outliers form the target zone compared to CTKA. With no other comprehensive RCT evaluating RATKA with the ROSA® knee system are available yet, the present study addresses an important gap in the literature and also provides a high level of evidence.

The present study found that postoperative patient‐reported outcomes are quite similar between RATKA and CTKA. This is in contrast to recently published data comparing RATKA using the ROSA® knee system to CTKA in a prospective matched comparative cohort study. The authors found significantly higher patient‐reported outcomes at 6‐month postoperative without differences in patient satisfaction rates compared to CTKA [20]. However, this study lacks of a randomised study design, leading to risk of patient selection bias which may be a potential reason for differences in postoperative outcomes compared to the present randomised‐controlled trial. Overall, there are conflicting results regarding postoperative outcomes of RATKA compared to CTKA, even from large‐scale reviews. For instance, Agarwal et al. demonstrated in a systematic review and meta‐analysis from 2020 the outcome of robotic‐assisted TKA including two randomised controlled trials and twenty cohort studies, encompassing over 2300 knees. Twelve studies found statistically better clinical outcomes in favour of RATKA compared to CTKA, while nine studies found no significant difference between the two techniques [1]. Otherwise, Alrajeb et al. included seven RCTs comparing RATKA to conventional TKA, encompassing approximately 1900 knees and found no statistically significant differences regarding postoperative outcomes between RATKA and CTKA [2]. It becomes clear, that with various robotic systems on the market, the direct comparison of clinical outcomes remains limited. Therefore, high‐level RCTs are needed to report on clinical outcomes for the most frequently used robotic systems. Therefore, the present study improves the evidence on postoperative outcomes using the ROSA® knee system. Even when not showing additional clinical benefit from RATKA in terms of short‐term PROMs the present findings demonstrated that, in subgroup analyses, variables such as age, sex and BMI do not significantly influence short‐term patient outcomes following TKA. Furthermore, small differences between the groups may be masked by a potential ceiling effect of the OKS. While it is a sensitive PROM to differentiate good from bad outcomes, it became incapable to distinguish good from excellent outcomes once a certain threshold is reached [6, 13].

Besides comparable patient‐reported outcomes, the present study found a significant higher surgical accuracy regarding postoperative limb alignment when performing RATKA by the ROSA® knee system. Postoperative limb alignment was already frequently analysed in several studies on RATKA. In line with the current results, significant less alignment outliers from the target zone were reported in a recent review report [1]. Therefore, superior referencing, precise bone cuts and knee balancing may be beneficial for precise limb alignment when using RATKA systems. For the ROSA® knee system, a more accurate restoration of joint line height and posterior condylar offset compared to CTKA has been reported in a retrospective case‐ control study [9]. However, improved surgical accuracy when using robotic assistants resulted in significant longer surgical duration. This increase is primarily attributed to the additional intraoperative steps required for robotic registration and anatomical mapping, which enable the system to recognise the patient′s knee structures [17, 23, 27]. While this might suggest a potential for increased blood loss, several other studies indicate no significant difference in blood loss between RATKA and CTKA [22, 25, 27]. In our findings there was a statistically significant difference in haemoglobin reduction, yet the difference of 0,3 mmol/l without need of blood transfusion in any included patient. Consequently, the present study improves the evidence regarding improved postoperative radiographic outcomes using the ROSA® knee system in a randomised‐controlled trial and showed no adverse effects of longer surgical duration while using a robotic system to assist in TKA.

However, even though this study compares CTKA and RATKA in a prospective RCT using the ROSA® knee system, there are several limitations to discuss. First, the number of patients in each cohort is relatively small, limiting the generalisability of the study′s results. A preoperative power analysis for a balanced one‐way analysis of variance (ANOVA) of the present study indicated an optimal number of 204 patients for each cohort in order to detect significant differences. However, such a high patient volume was not feasible due to practical and financial limitations. As the present study included a low number of patients, yet significant intergroup differences for patient‐reported outcomes might not be detected. Second, this study focused on the ROSA® knee system, which represents only one robotic system across numerous robotic systems available on the market. However, it is a frequently used robotic system on the marked and data regarding outcomes of this system are of critical importance for many clinicians. Furthermore, this study was not blinded—both surgeon and patients were aware of the surgical group assignment. Blinding of the surgeon was impossible since he designed the study and performed all operations. Yet, as a fellowship‐trained high‐volume surgeon there was no intention of different treatment of either group and the study was conducted independent from company sponsoring. Also, each patient was postoperatively aware of the type of TKA performed, as additional incisions were made for the placement of tibial trackers in the RATKA group. This may have led to subjective score deviations due to higher expectations for new technology and consequently distorting PROMs. Sham incisions in the CTKA group may have prevented this patient‐level bias. However, as these incisions are unnecessary for CTKA and could cause potential complications they are ethically not feasible.

Although follow‐up periods were statistically shorter in the robotic group (median difference less than 1 month), both groups were assessed close to the standard 1‐year postoperative time point. This small difference is unlikely to bias the comparison of functional outcomes.

As mentioned above, there was a patient selection bias as PS bearing was preferred to patients with limited knee flexion.

Despite the moderate variance explained by the model (*R*² = 0.36), the relatively small sample size may have limited the power to detect smaller effects. Also, the regression analyses may be subject to overfitting. However, the consistency of results between the reduced and full model supports the robustness of the findings. Further studies with larger cohorts and longer follow‐up periods are needed to validate these findings and explore potential interactions among covariates.

## CONCLUSION

Overall, this study demonstrates improved implant positioning and radiological alignment in RATKA with the ROSA® knee system compared to CTKA. Although robotic assistance shows similar patient‐reported outcomes compared to CTKA, the potential benefits in surgical precision and alignment could lead to improved long‐term outcomes and lower revision rates. Therefore, comprehensive long‐term RCTs are needed to assess the effects of RATKA on implant survival, revision rates as well as patient outcomes.

## AUTHOR CONTRIBUTIONS

Johannes Christian Reichert designed the study. Alexander Möller was involved in recruitment of study participants as well as clinical data collection. Johannes Christian Reichert, André Hofer and Georgi Iwan Wassilew supported and/or supervised clinical data collection. Johannes Christian Reichert, as senior knee arthroplasty surgeon, performed all operations. Alexander Möller, Feras Kasabji and Janosch Schoon analysed the clinical data and Janosch Schoon prepared the figures. Alexander Möller, Feras Kasabji and Maximilian Fischer wrote the main article. All authors critically reviewed and edited the manuscript and approved the manuscript.

## CONFLICT OF INTEREST STATEMENT

Georgi Iwan Wassilew takes a leadership role in Arbeitsgemeinschaft Endoprothetik (AE Germany), which is unrelated to this work. The remaining authors declare no conflict of interests. The named companies did not financially support this study, had no role in study design, sample collection, data collection and analysis, decision to publish, or preparation of the manuscript.

## ETHICS STATEMENT

Following approval by the ethics committee of the University Medicine Greifswald (BB 045/23), we conducted this prospective RCT in accordance with the most recent iteration of the World Medical Association Declaration of Helsinki between July 2022 and November 2024. All study participants provided written informed consent.

## Data Availability

All data generated or analysed in the course of this study are included in this published article.
